# Scar-concealed 2 + 3 mm dual-port thoracoscopic sympathectomy for palmar hyperhidrosis: single-center outcomes

**DOI:** 10.3389/fsurg.2025.1664901

**Published:** 2025-10-24

**Authors:** Qingjie Yang, Qingtian Li, Shenghua Lv, Linhui Lan, Ningquan Liu, Mingyang Wang, Xiaoyan Sun, Kaibao Han

**Affiliations:** Department of Thoracic Surgery, Xiamen Humanity Hospital, Fujian Medical University, Xiamen, China

**Keywords:** primary palmar hyperhidrosis, pinhole thoracoscope, sympathectomy, inconspicuous scar surgery, minimally invasive

## Abstract

**Objective:**

To minimize the trauma and incision of the operation for primary palmar hyperhidrosis (PPH), we have designed a inconspicuous scar thoracoscopic bilateral thoracic sympathetic chain transection via “2 + 3 mm” two-pinhole incisions (ISTTST). This study mainly retrospectively compares and analyzes the pros and cons of this surgical method vs. the conventional single-port thoracoscopic sympathetic nerve transection (CSTTST).

**Methods:**

Data of patients with moderate or severe PPH and underwent thoracic sympathetic chain transection were collected. Patients undergoing ISTTST and those receiving CSTTST were included in the two-pinhole group and the single-port group respectively. The baseline characteristics, intraoperative and postoperative conditions of the two groups were compared.

**Results:**

A total of 265 patients were enrolled, including 162 in the single-port group and 103 in the two-pinhole group. There were no statistically significant differences in baseline conditions such as gender, age, BMI, age of onset of PPH, hyperhidrosis sites, hyperhidrosis degree, and transection level of thoracic sympathetic chain between the two groups (*P* > 0.05). The two-pinhole group had shorter operation time (19.809 ± 3.356 min vs. 22.534 ± 4.541 min), lower postoperative incision pain score (1.563 ± 0.518 vs. 2.012 ± 0.788), and better incision satisfaction (9.437 ± 0.498 vs. 8.068 ± 1.424) (all *P* < 0.001). There were no statistically significant differences in postoperative conditions such as surgical effect, 24-h postoperative discharge rate, postoperative complication rate, postoperative compensatory hyperhidrosis, postoperative recurrence rate of PPH, and postoperative follow-up time between the two groups (*P* > 0.05).

**Conclusion:**

The ISTTST is a more concealed-scar, minimally invasive, and convenient procedure, meeting the aesthetic needs. Compared with the CSTTST, it has certain advantages and deserves more attention and attempts.

## Introduction

Thoracoscopic bilateral thoracic sympathetic chain transection is the most commonly used surgical method for the treatment of primary palmar hyperhidrosis (PPH). It is also the option when various antiperspirant methods such as topical antiperspirants, oral antiperspirants, electrolysis, botulinum, and computed tomography guided puncture with absolute alcohol/microwave ablation/radiofrequency ablation to damage the sympathetic nerve chain are ineffective (1–4). Its therapeutic effect has been proven to be exact and lasting (4–6). In recent years, there have been few studies on thoracoscopic thoracic sympathetic chain transection/endoscopic thoracic sympathicotomy for the treatment of PPH. They mainly focus on which level of the sympathetic nerve chain should be transected during the surgery (one or several of T2, T3, T4, and T5) (7, 8), the prediction and prevention of postoperative compensatory hyperhidrosis (9, 10), as well as the improvement of surgical procedures. The surgical procedures for palmar hyperhidrosis are basically developing in the direction of minimally invasive, rapid recovery, and incision cosmesis. For example, surgeries without tracheal intubation/laryngeal mask (11, 12), single-port surgeries, and surgeries through the areola (13, 14), etc. Among them, single-port and two-port thoracoscopic bilateral thoracic sympathetic chain transection are the surgical methods most commonly adopted by clinicians at present. Both methods have their advantages and disadvantages. The incision of single-port surgery is more aesthetic, but it is relatively difficult to operate the endoscope and electrocautery hook within the same small incision during the surgery; the two-port surgery is relatively easier to operate, but with one more incision, the aesthetic effect is poorer.

Based on years of conducting sympathetic nerve surgeries in our team (including bilateral thoracic sympathetic chain transection for palmar hyperhidrosis, and transection of greater and lesser splanchnic nerves for upper abdominal cancer pain, etc.), and considering the advantages and disadvantages of single-port and two-port thoracoscopic surgeries, we have designed a more concealed-scar, convenient to operate, and highly safe “inconspicuous scar thoracoscopic bilateral thoracic sympathetic chain transection via ‘2 + 3 mm’ two-pinhole incisions (ISTTST)”. Since 2018, more than 100 cases of this surgery have been performed. In this article, we will introduce this surgical method and retrospectively compare and analyze its advantages and disadvantages compared with the conventional single-port thoracoscopic bilateral thoracic sympathetic chain transection (CSTTST), as well as the results of long-term follow-up via WeChat.

## Methods

### Ethics statement

This study was approved by the Medical Ethics Committee of Xiamen Humanity Hospital of Fujian Medical University (NO. HAXM-EMC-20221017-001-01). This study was in accordance with the provisions of the Declaration of Helsinki. Considering its retrospective design, the requirement of informed consent of each patient was waived by the ethics committee.

### Cases collected

This study enrolled patients with moderate or severe PPH who received surgical treatment at Xiamen Humanity Hospital of Fujian Medical University from October 1, 2018 to September 30, 2023. The diagnostic criteria, severity classification, surgical indications and contraindications for PPH were carried out in accordance with the *Expert Consensus On Minimally Invasive Treatment Of Palmar Hyperhidrosis In China* (15).

Case inclusion criteria: Patients who underwent CSTTST or ISTTST due to moderate or severe PPH. Exclusion criteria: ① patients with mild palmar hyperhidrosis who mainly visited for surgery due to craniofacial hyperhidrosis; ② patients with significantly prolonged operation time or changed surgical procedure due to pleural adhesions; ③ patients who did not cooperate with follow-up or were lost to follow-up after surgery.

Because the 2.7 mm endoscope we used during the surgery was borrowed from the gynecology department. They routinely use the 2.7 mm endoscope for hysteroscopy surgeries. According to hospital regulations, the gynecology department has priority in using the 2.7 mm endoscope. Therefore, if the gynecologists do not use the 2.7 mm endoscope on the surgery day, we perform ISTTST. When the gynecologists need to use the 2.7 mm endoscope, we perform the CSTTST. Thus, patients undergoing palmar hyperhidrosis surgery at our hospital were passively divided into the two-pinhole group (receiving ISTTST) or the single-port group (receiving CSTTST).

### Special surgical instrument

#### 1.8 mm electrocautery hook

**Figure 1:**
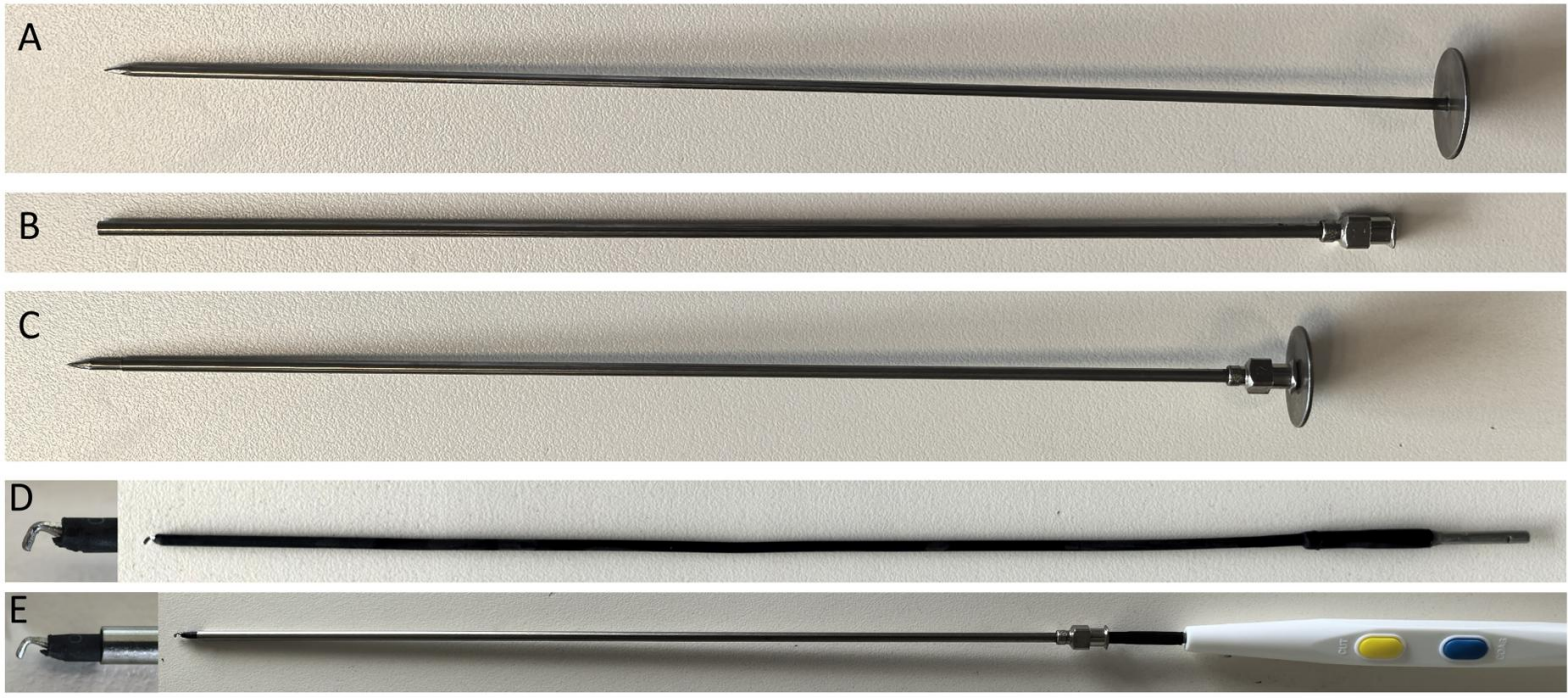
1.8 mm electrocautery and its matching trocar. **(A)** Inner core of the Trocar; **(B)** Outer sheath of the Trocar; **(C)** Assembled state of the Trocar; **(D)** electrocautery hook; **(E)** The electrocautery hook is loaded into the electrocautery handle and passed through the Trocar.

It is a slender electrocautery hook with a diameter of 1.8 mm and a length of 23 cm. The head is hook-shaped, and the tail 2 cm is bare metal, which can be attached to the conventional high-frequency electrocautery handle for surgery. The matching Trocar is made of 304 stainless steel, with an outer diameter of 2 mm and a length of 20 cm. When using it, first install the inner core of the Trocar, and puncture the Trocar into the thoracic cavity through the skin, then withdraw the inner core. Introduce the 2 mm electrocautery hook from the Trocar. When fully introduced, the head of the electrocautery hook will protrude 1 cm from the Trocar. Because the 1.8 mm electrocautery hook is relatively thin and prone to bending and shaking, when using it, the Trocar and the electrocautery hook are moved as one to increase the stability of the operation. The 1.8 mm electrocautery hook and matching 2 mm Trocar were custom-manufactured by Xiame Jiayou Co., Ltd. (Fujian, China). see Figure 1.

#### 2.7 mm endoscope

The 30-degree endoscope we used is the one used in hysteroscopy surgeries in gynecology, with an outer diameter of 2.7 mm (Karl Storz, Tuttlingen, Germany). The matching Trocar has an outer diameter of 3 mm and is equipped with an inflation interface (Kangji Medical Instrument Co., Ltd, China).

#### Inconspicuous scar thoracoscopic bilateral thoracic sympathetic chain transection via “2 + 3 mm” two-pinhole incisions (ISTTST)

**Figure 2:**
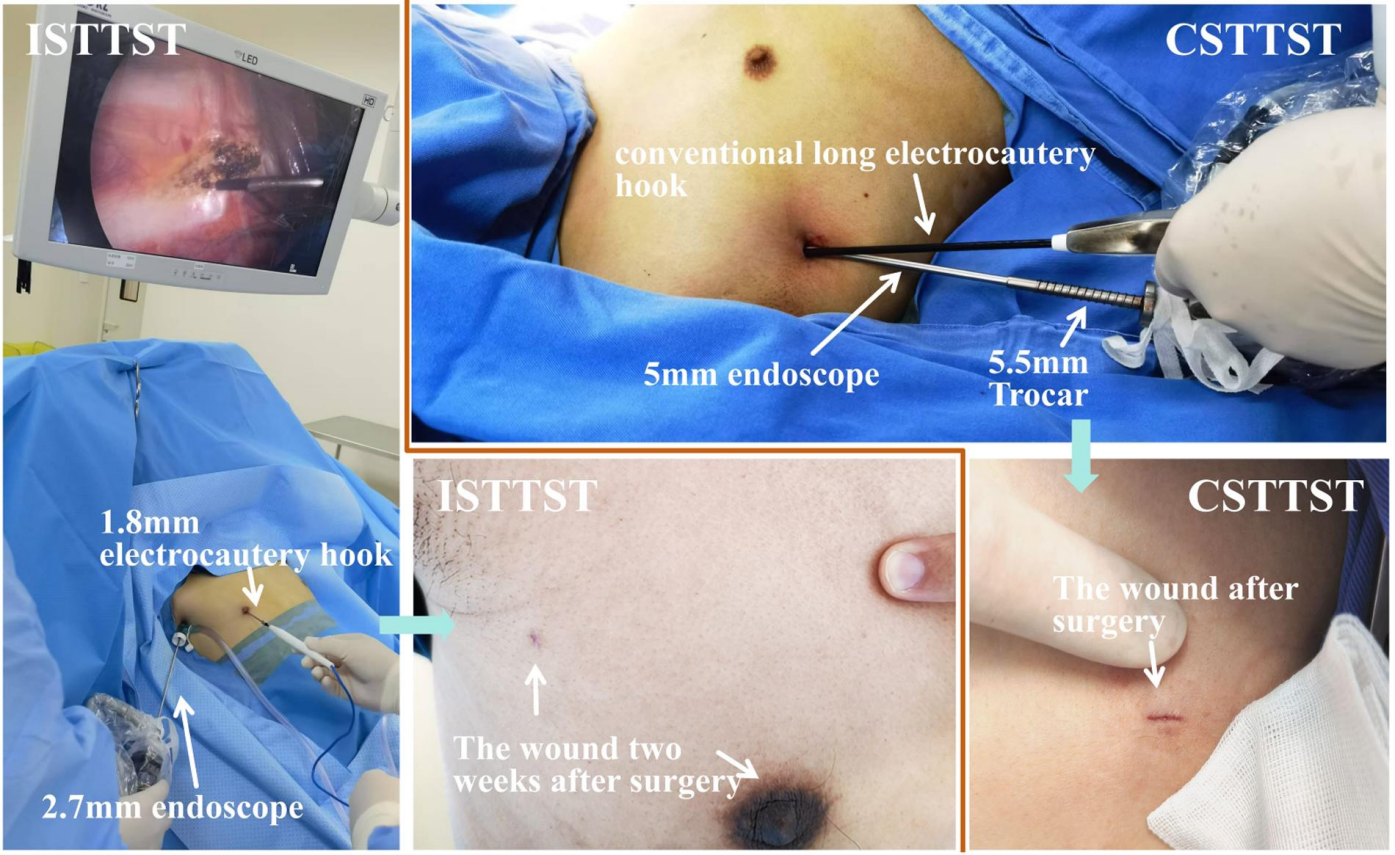
The operation methods of the two surgical procedures and the postoperative incisions. ISTTST: Inconspicuous scar thoracoscopic bilateral thoracic sympathetic chain transection via "2+3 mm" two-pinhole incisions. CSTTST: Conventional single-port thoracoscopic bilateral thoracic sympathetic chain transection.

Intravenous anesthesia was administered, and a laryngeal mask was inserted. The patient was placed in a 45° semi-recumbent position, with both hands abducted, the back padded up, and bilateral axillary regions fully exposed. Generally, the right side was operated on first, followed by the left side. During the operation, low tidal volume ventilation was adopted, with a tidal volume of approximately 300 ml. A 3 mm skin incision was made at the 3rd intercostal space of the anterior axillary line, and a 3 mm diameter Trocar was placed into the thoracic cavity. The Trocar was connected to the pneumoperitoneum machine, and CO_2_ was continuously blown into the thoracic cavity at a flow rate of 8 l/min to maintain a thoracic pressure of 8–10 mmHg, and a 2.7 mm endoscope was introduced. For male patients, a 2 mm incision was made on the skin at the areola, while for female patients, it was made at the lower margin of the breast. A 2 mm-diameter Trocar was inserted, and a 1.8 mm electrocautery hook was introduced. The situation inside the thoracic cavity was examined. If adhesions were observed within the thoracic cavity, the electrocautery hook was utilized to release the adhesions, and the positions of the T3/T4 sympathetic nerve chains were fully exposed and confirmed. The sympathetic nerve chain was horizontally transected on the surface of the 3rd or 4th rib using the electrocautery hook, ensuring that the distance between the upper and lower broken ends was greater than 8 mm. Additionally, the areas 1–2 cm on the left and right sides of the sympathetic nerve chain along the rib surface were cauterized. Confirm that there is no bleeding in the surgical field. The electrocautery hook was withdrawn, and the 2 mm Trocar was extended to the vicinity of the thoracic roof. The pneumoperitoneum was halted, the 2 mm Trocar was connected to negative pressure suction, and after observing satisfactory lung re-expansion under the endoscopy, the endoscope and the 3 mm Trocar were removed. Finally, the 2 mm Trocar was withdrawn while maintaining the connection to negative pressure suction. The two incisions were respectively bonded with medical glue. The contralateral thoracic sympathetic nerve chain transection was carried out in the same manner see Figure 2.

### Conventional single-port thoracoscopic bilateral thoracic sympathetic chain transection (CSTTST)

The anesthesia, laryngeal mask, recumbent position, disinfection, and low tidal volume ventilation were all the same as those in the two-pinhole group. A 6 mm small incision was made on the skin at the 3rd intercostal space of the anterior axillary line. The anesthesiologist was instructed to temporarily disconnect the ventilator and open the laryngeal mask pipeline. A 5.5 mm-diameter Trocar was held and punctured into the thoracic cavity. After the formation of artificial pneumothorax, a 5 mm 30-degree endoscope was introduced to confirm the location of the T3/T4 sympathetic nerve trunk. Keeping the thoracoscope stationary, the Trocar was withdrawn from the incision (at this time, the Trocar was still sleeved on the thoracoscope), and an electrocoagulation hook was introduced from the incision, closely adhering to the endoscope. The sympathetic nerve chain was transected on the surface of the 3rd/4th rib, ensuring that the distance between the upper and lower broken ends was >8 mm, and the area 1–2 cm on the outside of the sympathetic nerve trunk along the rib surface was cauterized. After a satisfactory resection, the electrocoagulation hook was withdrawn, the Trocar was pushed back into the incision, the thoracoscope was withdrawn, a 16G suction catheter was introduced from the Trocar, the suction catheter was kept unmoved, and the Trocar was withdrawn. The end of the suction catheter was placed in water, the lungs were inflated to expel gas, and the suction catheter was removed after the accumulated gas in the thoracic cavity was discharged through the suction catheter. The incision was bonded with medical glue. The contralateral thoracic sympathetic nerve chain transection was performed in the same manner see Figure 2.

### Postoperative care

The patient regained consciousness approximately 20 min after the surgery and was observed in the recovery room for about half an hour before being sent back to the ward. The patient was observed in the ward for 4–6 h after the surgery. If the re-examination of the chest radiograph revealed no obvious hydropneumothorax and the patient had no significant discomfort, they could be discharged home. If the surgery was conducted in the afternoon, they would be discharged home the next morning.

### Follow-up

All patients were followed up at the outpatient department respectively at 2 weeks and 1 month after the surgery, and were followed up by Wechat one year after the surgery. The main points to be investigated included the healing status of the surgical incision, the antiperspirant effect after the surgery, the satisfaction with the aesthetic appearance of the surgical incision, whether there were complications such as compensatory hyperhidrosis, and whether the palmar hyperhidrosis recurred, etc.

### Obvervational index

The observational indices include: gender, age at surgery, BMI (Body Mass Index), age of onset of palmar hyperhidrosis, hyperhidrotic sites, degree of hyperhidrosis, level of thoracic sympathetic chain transection, operation time, whether discharged within 24 h after surgery, postoperative complications, highest postoperative pain score (POPS), antiperspirant effect, satisfaction with the surgical incision, compensatory hyperhidrosis, recurrence of palmar hyperhidrosis, and postoperative follow-up time. Since intraoperative blood loss was mostly less than 5 ml and difficult to measure accurately, intraoperative blood loss was not included as an observational index. The definitions of some observational indices are as follows.

Hyperhidrotic Sites: There are several combinations of hyperhidrotic sites for patients with PPH. They are only hands, hands + axillae, hands + feet, hands + feet + axillae, hands + head + axillae, hands + head + feet + axillae.

Degree of Hyperhidrosis: According to the *Expert Consensus On Minimally Invasive Treatment Of Palmar Hyperhidrosis In China* (15), it is divided into 3 degrees: Mild—Moist hands, Moderate—Wet hands with visible sweating drops, Severe—Very wet hands with dripping sweating.

Level of Thoracic Sympathetic Chain Transection: Generally, it is T3 or T4. Because compensatory hyperhidrosis is more obvious after transecting T3, if T3 is transected on one side and R4 on the other side, it is recorded as T3.

Postoperative Complications: Such as pneumothorax, hemothorax, pulmonary or thoracic infections, Horner's syndrome, poor incision healing, etc.

Highest POPS: The highest pain score obtained from multiple assessments during the interval between the end of surgery and discharge; the assessments were performed twice daily and during each episode of intense pain using a visual analogue scale.

Antiperspirant Effect: According to the degree of reduction in palmar hyperhidrosis after surgery, it is divided into 5 grades (see Table 1).

**Table 1:** Grading standards of some observational indicators.

Evaluation indicator	Grade 0	Grade 1	Grade 2	Grade 3	Grade 4
Antiperspirant Effect	Surgery ineffective, no reduction in palmar hyperhidrosis after surgery	Under the same circumstances, the palmar sweating after surgery decreased by <25% compared with that before surgery	Under the same circumstances, the palmar sweating after surgery decreased by 25%–75% compared with that before surgery	Under the same circumstances, the palmar sweating after surgery decreased by >75% compared with that before surgery	After surgery, both hands are warm and without sweating Compensatory Hyperhidrosis
Compensatory hyperhidrosis	No compensatory hyperhidrosis	The skin is moist, without hyperhidrosis or any discomfort.	There is obvious sweating and discomfort, but it can be tolerated.	Excessive sweating, the sweat can flow, and the clothes need to be changed multiple times a day due to hyperhidrosis, but it can be tolerated and the patient does not regret the surgery.	Excessive sweating, the sweat can flow, seriously affecting the quality of normal life, intolerable, and the patient regrets the surgery.

Satisfaction with the Surgical Incision: After the surgical incision healed, patient satisfaction with incision aesthetics was evaluated using a 10-point visual analogue scale (VAS) modeled after the validated pain VAS system. Patients scored their satisfaction based on two parameters: 1. scar concealment (visibility at conversational distance) and 2. aesthetic integration (color/texture match with surrounding skin). This approach aligns with ISO-20031:2020 recommendations for patient-reported scar evaluation (16), where 1 = “extremely dissatisfied” (clearly visible hypertrophic scar) and 10 = “extremely satisfied” (imperceptible scar at 50 cm distance). While formal validation for sympathetic surgery contexts is pending, this method provides clinically actionable data reflecting patient priorities.

Compensatory Hyperhidrosis: The degree of postoperative compensatory hyperhidrosis was evaluated according to Tu's 5-level method (see Table 1).

Recurrence of Palmar Hyperhidrosis: After the surgery, for one or both hands, the palmar hyperhidrosis first decreased and then increased to be similar to that before the surgery, and lasted for more than 3 months.

### Statistical analysis

Given the exploratory nature of this study comparing a novel technique, formal *a priori* sample size calculation was not performed. However, a *post hoc* power analysis using PASS 15.0 (NCSS, LLC, Kaysville, UT) was conducted for the three primary outcomes: operative time, pain score, and incision satisfaction. Based on the observed effect sizes (Cohen's *d* = 0.661 for operative time, 0.645 for pain score, and 1.184 for satisfaction) and actual sample sizes (162 vs. 103), the study achieved >99.9% power for all outcomes at *α* = 0.05 (two-sided), far exceeding the conventional 80% threshold. This confirms the adequacy of our sample size to detect clinically meaningful differences. SPSS version 26.0 (IBM Corp, USA) was used for all statistical analyses. Normally distributed data were presented as means (SD: standard deviation), while non-normally distributed data were represented by the medians [IQRs (interquartile ranges): first quartile—third quartile]. Categorical data were presented as numbers with percentages. Normally distributed continuous data were analyzed using independent samples t test. Non-normally distributed continuous data were analyzed using the independent samples Mann–Whitney *U* test. Categorical data were analyzed using the chi-square test or Fisher's exact test, which was used when the expected frequency was less than 5%. All statistical analyses were conducted with an *α* cut-off value of 0.05.

## Results

A total of 265 patients were enrolled, including 162 cases in the single-port group and 103 cases in the two-pinhole group. There were no statistically significant differences in baseline conditions such as gender, age, BMI, age of onset of palmar hyperhidrosis, hyperhidrotic sites, degree of hyperhidrosis, and level of thoracic sympathetic chain Transection between the two groups (*P* > 0.05).

The operation time of the two-pinhole group was shorter than that of the single-port group [19.81 ± 3.36, 95% CI (19.29, 20.33) vs. 22.53 ± 4.54, 95% CI (21.65, 23.42), *P* < 0.001], the postoperative incision pain score was lower than that of the single-port group [1.56 ± 0.52, 95% CI [1.46, 1.66] vs. 2.01 ± 0.79, 95% CI (1.89, 2.13), *P* < 0.001], and the postoperative incision satisfaction was better than that of the single-port group [9.44 ± 0.50, 95% CI [9.34, 9.53] vs. 8.07 ± 1.42, 95% CI [7.85, 8.29], *P* < 0.001].

There were no statistically significant differences between the two groups in terms of surgical effect, discharge rate within 24 h after surgery, incidence of postoperative complications, postoperative compensatory hyperhidrosis, recurrence rate of palmar hyperhidrosis after surgery, and postoperative follow-up time (*P* > 0.05) (see Table 2).

**Table 2:** Comparison of observational indicators between the Two groups of patients.

Observational indicators	Subcategory	Single-port group (*n* = 162)	Two-pinhole group (*n* = 103)	*t*/*χ*^2^****/*Z* value	*P* value
Gender, *n* (%)	Male	86 (53.09%)	45 (43.69%)	2.224	0.136
	Female	76 (46.91%)	58 (56.31%)		
Age at Surgery (year), Mean ± SD*, [95% CI]**		22.93 ± 6.60 [21.90, 23.95]	24.09 ± 6.97 [22.72, 25.45]	−1.366	0.173
BM^#^, Mean ± SD, [95% CI]		21.22 ± 5.63 [20.35, 22.10]	20.34 ± 2.07 [19.94, 20.75]	1.527	0.128
Age of onset of PPH## (year), Mean ± SD, [95% CI]		9.01 ± 3.94 [8.39, 9.62]	9.46 ± 3.27 [8.82, 10.10]	−0.966	0.335
Hyperhidrotic sites, *n* (%)	Only hands	12 (7.41%)	3 (2.91%)	6.594	0.086
	Hands + axillae	83 (51.23%)	47 (45.63%)		
	Hands + feet + axillae	64 (39.51%)	53 (51.46%)		
	Hands + feet	3 (1.85%)	0 (0%)		
Degree of hyperhidrosis, *n* (%)	Moderate	70 (44.44%)	33 (32.04%)	−1.815	0.070
	Severe	92 (56.79%)	70 (67.96%)		
Level of thoracic sympathetic chain transection, *n* (%)	T3	149 (88.27%)	93 (90.29%)	0.225	0.635
	T4	13 (8.02%)	10 (9.71%)		
Operation time (min), Mean ± SD, [95% CI]		22.53 ± 4.54 [21.65, 23.42]	19.81 ± 3.36 [19.29, 20.33]	−5.604	<0.001
Highest postoperative pain score, Mean ± SD, [95% CI]		2.01 ± 0.79 [1.89, 2.13]	1.56 ± 0.52 [1.46, 1.66]	5.124	<0.001
Antiperspirant effect, *n* (%)	Grade 2	1 (0.62%)	0 (0%)	−0.089	0.929
	Grade 3	4 (2.47%)	3 (2.91%)		
	Grade 4	157 (96.91%)	100 (97.09%)		
Postoperative complications, *n* (%)	No	155 (95.68%)	102 (99.03%)	2.489	0.288
	Pneumothorax	6 (3.70%)	1 (0.97%)		
	Poor incision healing	1 (0.62%)	0 (0%)		
Discharged within 24 h after surgery, *n* (%)	Yes	156 (96.30%)	102 (99.03%)	1.829	0.253
	No	6 (3.70%)	1 (0.97%)		
Compensatory hyperhidrosis, *n* (%)	Grade 0	6 (3.70%)	2 (1.94%)	−0.449	0.653
	Grade 1	35 (21.60%)	29 (28.16%)		
	Grade 2	63 (38.89%)	36 (34.95%)		
	Grade 3	58 (35.80%)	36 (34.95%)		
	Grade 4	0	0		
Recurrence of palmar hyperhidrosis, *n* (%)	Yes	1 (0.62%)	1 (0.97%)	0.105	1.000
	No	161 (99.38%)	102 (99.03%)		
Satisfaction with the surgical incision, Mean ± SD, [95% CI]		8.07 ± 1.42 [7.85, 8.29]	9.44 ± 0.50 [9.34, 9.53]	−9.395	<0.001
Postoperative follow-up time (month), Median (IQRs***: First Quartile, Third Quartile)		35.50 (IQRs: 22.00–49.00)	35.00 (IQRs: 25.00–44.00)	−0.262	0.793

* SD, standard deviation.

** 95% CI, 95% Confidence Interval.

*** IQRs, interquartile ranges.

**** *χ*^2^, chi-square test.

# BMI, Body Mass Index.

## PPH, primary palmar hyperhidrosis.

In the single-port group, 5 patients (3.09%) had a 0.5 cm small incision added at the 4th intercostal space on the anterior rib line on one side due to intraoperative bleeding from the surgical field and difficult exposure of the surgical area. There were no patients with additional surgical incisions in the two-pinhole group. None of the patients in both groups had their surgical incisions extended during the operation. Among the postoperative complications, in the single-port group, 6 patients (3.70%) had postoperative pneumothorax, all of which were unilateral pneumothorax. Among them, 3 cases had a large amount of pneumothorax, with lung compression greater than 30%, and improved after puncture and aspiration of gas from the 2nd intercostal space of the midclavicular line. The other 3 cases had a small amount of pneumothorax and were absorbed spontaneously. In the two-pinhole group, 1 patient (0.62%) had a small amount of postoperative pneumothorax, and no special treatment was given and it was absorbed spontaneously. In the single-port group, 1 patient (0.62%) had poor healing of the surgical incision on the left thoracic wall after the operation and healed after dressing change.

## Discussion

Thoracoscopic bilateral thoracic sympathetic nerve chain transection is one of the most effective methods for treating PPH (17). Although the trauma of this surgery is already very small, for a disease like palmar hyperhidrosis that has a relatively small impact on physical health, undergoing intrathoracic surgery still makes most patients hesitate. Therefore, the craftsmanship spirit of thoracic surgeons has been fully exerted in such “minor surgeries” as thoracic sympathetic nerve chain transection. The surgeries have become increasingly minimally invasive, and the incidence of complications has also been well controlled.

Thoracoscopic thoracic sympathetic nerve chain transection was initially a three-port thoracoscopic surgery. The advantage was that the surgical operation was convenient, and complications such as bleeding during the operation could be dealt with in time without the need to add incisions temporarily. The disadvantage was that there were multiple incisions and obvious postoperative pain. In the 34 cases of PPH treated with the three-port thoracoscopic method reported by Shioe et al. (18), in addition to pain, 50% of the patients presented with chest wall paresthesia mainly characterized by contractions, needle-like sensations, or numbness. Post-thoracoscopic postoperative pain was relieved in most patients within 2–4 weeks, while chest wall paresthesia sometimes persisted for more than 12 months. Mechanical injury to the intercostal nerves might be the main reason (19, 20). With the improvement of surgical techniques, the surgical incision of thoracoscopic thoracic sympathetic nerve chain transection has gradually decreased to two ports, and even to a single port of 0.5–1 cm. Currently, single-port thoracoscopic surgery is more commonly used. The advantage is that there are fewer surgical incisions and less postoperative pain. The disadvantage is that the thoracoscope and energy devices are introduced through the same small hole, and the devices operating in the same direction are prone to mutual interference, which is not conducive to the exposure and fine resection of the sympathetic nerve (15, 21). Some doctors also perform two-port thoracoscopic surgery through areolar incisions. One surgical incision is under the armpit and the other is on the areola. The advantages are two incisions, convenient surgical operation, and the surgical incisions are concealed, meeting the aesthetic requirements, especially the incision on the areola is almost invisible after healing. The disadvantage is that this surgical method is only suitable for male patients, while women are the group with a high demand for aesthetic incisions (13, 14).

Interestingly, despite the availability of single-port thoracoscopic surgery, both palmar hyperhidrosis patients and thoracic surgeons still have higher expectations. Patients desire more aesthetic incisions and fewer complications. Surgeons aim to complete the surgery through smaller incisions and make the operation more manageable. Against this backdrop, our team has carried out the inconspicuous scar thoracoscopic bilateral thoracic sympathetic chain transection via “2 + 3 mm” two-pinhole incisions. This surgical method has the following advantages: 1. compared with single-port surgery, during two-pinhole surgery, the thoracoscope and the electrocautery hook reach the surgical area from different directions. The thoracoscope and the electrocautery hook do not interfere with each other, making exposure easier, allowing for closer observation of the sympathetic nerve, smoother operation, and shorter operation time (19.809 ± 3.356 vs. 22.534 ± 4.541, *P* < 0.001). 2. In two-pinhole surgery, because the Trocar is retained on the chest wall, the pneumoperitoneum machine can be used to inflate the thoracic cavity, causing the lung tissue to collapse and creating a better surgical field. It is very convenient to remove smoke from the thoracic cavity and wipe the thoracoscopic lens during the operation. However, during single-port surgery, the Trocar needs to be withdrawn from the incision, leaving only the thoracoscope and the electrocautery hook in the incision. Inflation of the thoracic cavity through the pneumoperitoneum machine is not possible, and both the collapse of the lung tissue and the exposure of the surgical field are not as good as in two-pinhole surgery. During single-port surgery, if it is necessary to remove smoke from the thoracic cavity or wipe the thoracoscopic lens, the electrocautery hook needs to be withdrawn, the Trocar reinserted, and when performing the resection operation, the Trocar is withdrawn again and the electrocautery hook inserted. This not only complicates the operation but also increases the trauma to the incision. 3. Compared with single-port thoracoscopic surgery, in two-pinhole surgery, due to the tiny incisions and the absence of repeated insertion and withdrawal of the Trocar and the electrocautery hook during the operation, the interference with the chest wall tissue is small, the postoperative wound pain is less, and the highest postoperative incision pain score is significantly lower than that of single-port surgery (1.563 ± 0.518 vs. 2.012 ± 0.788, *P* < 0.001). 4. After two-pinhole surgery, the incision does not require suturing and can be bonded with medical glue. The incision is aesthetic after healing, and the wound is almost invisible after about 1 month, achieving the effect of inconspicuous scarring. Patients who underwent two-pinhole surgery have significantly higher satisfaction with the incision (9.437 ± 0.498 vs. 8.068 ± 1.424, *P* < 0.001); 5. at the end of two-pinhole surgery, the situation of thoracic cavity exhaust and lung re-expansion can be observed under the thoracoscope to avoid a large amount of residual gas in the thoracic cavity after the surgery. In single-port surgery, exhaust can only be performed through the drainage tube without monitoring. There are situations where the position of the drainage tube is not ideal or the lung compliance is poor, resulting in incomplete gas exhaust in the thoracic cavity during the operation, and the need for re-puncture and aspiration of gas after the operation. In this study, although there was no statistically significant difference in the incidence of postoperative complications between the two groups of patients, the incidence of postoperative pneumothorax in the two-pinhole group was numerically significantly lower than that in the single-port group (0.97% vs. 3.70%).

Despite the promising outcomes observed in this study, several limitations warrant consideration. First, the non-randomized allocation of patients, dictated solely by the availability of specialized instrumentation, may introduce selection bias and constrain the generalizability of comparative findings. Second, the retrospective observational design inherently limits causal inference regarding technique superiority. Third, while clinically pragmatic, the 10-point incision satisfaction scale utilized lacks formal validation, potentially affecting quantitative interpretation of cosmetic outcomes. Finally, the absence of *a priori* sample size calculation—though mitigated by *post hoc* power analysis—represents a methodological constraint.

## Conclusion

The inconspicuous scar thoracoscopic bilateral thoracic sympathetic chain transection via “2 + 3 mm” two-pinhole incisions is a more inconspicuous, minimally invasive, and convenient surgical method that caters to the aesthetic needs of palmar hyperhidrosis patients for surgical incisions. It deserves more attention and attempts for implementation.

## Data Availability

The original contributions presented in the study are included in the article/Supplementary Material, further inquiries can be directed to the corresponding author.
